# Succinobucol-Eluting Stents Increase Neointimal Thickening and Peri-Strut Inflammation in a Porcine Coronary Model

**DOI:** 10.1002/ccd.24473

**Published:** 2012-05-14

**Authors:** Jonathan Watt, Simon Kennedy, Christopher McCormick, Ejaife O Agbani, Allan McPhaden, Alexander Mullen, Peter Czudaj, Boris Behnisch, Roger M Wadsworth, Keith G Oldroyd

**Affiliations:** 1 Strathclyde Institute of Pharmacy and Biomedical Sciences, University of StrathclydeGlasgow, United Kingdom; 2 West of Scotland Regional Heart & Lung Centre, Golden Jubilee National HospitalGlasgow, United Kingdom; 3 University of Glasgow, Institute of Cardiovascular & Medical SciencesGlasgow, United Kingdom; 4 Department of Pathology, Glasgow Royal InfirmaryUnited Kingdom; 5 Translumina GmbHHechingen, Germany

**Keywords:** angioplasty, stents, antioxidants, restenosis, inflammation

## Abstract

**Objective:**

The aim of this study was to assess the efficacy of stent-based delivery of succinobucol alone and in combination with rapamycin in a porcine coronary model. *Background*: Current drugs and polymers used to coat coronary stents remain suboptimal in terms of long term efficacy and safety. Succinobucol is a novel derivative of probucol with improved antioxidant and anti-inflammatory properties.

**Methods:**

Polymer-free Yukon stents were coated with 1% succinobucol (SucES), 2% rapamycin (RES), or 1% succinobucol plus 2% rapamycin solutions (SucRES) and compared with a bare metal stent (BMS).

**Results:**

The *in vivo* release profile of SucES indicated drug release up to 28 days (60% drug released at 7 days); 41 stents (BMS, *n* = 11; SucES, *n* =10; RES, *n* = 10; SucRES, *n* = 10) were implanted in the coronary arteries of 17 pigs. After 28 days, mean neointimal thickness was 0.31 ± 0.14 mm for BMS, 0.51 ± 0.14 mm for SucES, 0.19 ± 0.11 mm for RES, and 0.36 ± 0.17 mm for SucRES (*P* < 0.05 for SucES vs. BMS). SucES increased inflammation and fibrin deposition compared with BMS (*P* < 0.05), whereas RES reduced inflammation compared with BMS (*P* < 0.05).

**Conclusion:**

In this model, stent-based delivery of 1% succinobucol using a polymer-free stent platform increased neointimal formation and inflammation following coronary stenting. © 2012 Wiley Periodicals, Inc.

## INTRODUCTION

Drug-eluting stents (DES) have decreased the incidence of in-stent restenosis (ISR) compared with bare-metal stents (BMS). However, polymers and drugs coated on stents can delay arterial healing and cause inflammation, which may increase the risk of stent thrombosis and late “catch-up” restenosis [1]. Thus, much effort is now being directed towards the development of novel DES which inhibit ISR and promote healing by reducing inflammation.

Preclinical studies have shown that oral probucol inhibits neointimal hyperplasia and improves re-endothelialization after stent injury via its antioxidant properties and up-regulation of heme oxygenase-1, which induces vascular smooth muscle cell (SMC) apoptosis and promotes endothelial cell (EC) function [2–5]. However, the effects of oral probucol have been disappointing in clinical trials [6–9]. The use of probucol as a stent coating has proven more valuable in a porcine coronary model [10] and subsequently in the large ISAR-TEST-2 (Intracoronary Stenting and Angiographic Results: Test Efficacy of three Limus-Eluting Stents) clinical trial, which showed that a polymer-free probucol/rapamycin-eluting stent provided improved efficacy compared with durable polymer-based rapamycin or zotarolimus-eluting stents, with lower rates of “catch-up” restenosis between one and two years [11,12].

Succinobucol is a novel derivative of probucol, which has consistently demonstrated improved pharmacokinetics and superior antioxidant, antiproliferative, and anti-inflammatory effects compared with probucol [13–16]. Succinobucol inhibits proinflammatory cytokine release by monocytes, expression of proinflammatory cell adhesion molecules by ECs [13–15] and platelet aggregation [17], all of which may contribute to restenosis. In the ARISE (Aggressive Reduction of Inflammation Stops Events) clinical trial, oral succinobucol reduced the incidence of myocardial infarction, stroke, and diabetes mellitus; however it brought about deleterious changes in lipid profiles and increased the incidence of atrial fibrillation [18]. In CART-1 (Canadian Antioxidant Restenosis Trial), oral succinobucol following BMS implantation reduced ISR, but only when drug compliant patients were analyzed separately [9]. It is possible that locally targeted succinobucol therapy achievable by stent-based delivery might improve efficacy and reduce systemic adverse effects. However, if negative cellular actions outweigh its beneficial effects, delivery of succinobucol into the artery wall from a stent may lead to localized toxicity. Bearing in mind the recognized negative effects of permanent polymers, this preclinical study was designed to test whether local delivery of succinobucol alone or in combination with rapamycin in the absence of a polymer would have a favorable effect on vascular healing after stent implantation.

## METHODS

### Drugs

Succinobucol (previously AGI-1067), the monosuccinic acid ester of probucol, was synthesized by esterification of probucol (Sigma-Aldrich, Poole, Dorset). The identity of the product was confirmed by NMR spectroscopy and purity was in excess of 99%. Succinobucol is metabolically stable and no significant active metabolites are formed *in vivo* [19]. Rapamycin (sirolimus) is a macrocyclic triene antibiotic with potent antiproliferative, anti-inflammatory, and immunosuppressive effects. It forms a complex with FKBP12, which subsequently binds to and inhibits the molecular target of rapamycin (mTOR), causing arrest of cell proliferation. Rapamycin (purity ≥95%) was purchased from Cfm Oskar Tropitzsch (Marktredwitz, Germany).

### DES Platform

The Yukon DES (Translumina, Hechingen, Germany) used in this study consisted of a pre-mounted, sandblasted 316L stainless steel microporous stent, which is designed for on-site stent coating without the obligate use of a polymer. The detailed process of stent coating and mechanical stent surface modification for increased drug storage capacity has been described in detail previously [20]. All stents used were 3.5 mm in diameter and 16 mm in length. BMS were uncoated versions of the Yukon stent. All coating solutions consisted of drug(s) dissolved in 99.5% ethanol. During bench testing, 0.5% (5 mg/ml), 1% (10 mg/ml), and 2% (20 mg/ml) succinobucol solutions were sprayed onto a Yukon® stent and closely examined using scanning electron microscopy (Hitachi S-4800). 1% succinobucol coating solution produced a superior, smooth, and uniform complete drug layer, optimal for the Yukon® DES delivery system and therefore was considered most appropriate for initial preclinical assessment. Three DES were investigated: a succinobucol-eluting stent (SucES) which utilized a 1% succinobucol coating solution; a rapamycin-eluting stent (RES) which utilized a 2% rapamycin coating solution; and a dual succinobucol/rapamycin-eluting stent (SucRES) which utilized a 1% succinobucol/2% rapamycin coating solution. All stents were coated within 24 hr of use. The coating concentration of rapamycin was derived from published data [20,21].

### Porcine Coronary Stent Model

Male large, white Landrace pigs (16–22 kg) were premedicated with aspirin (300 mg oral) and clopidogrel (300 mg oral), before sedation by an injection of tiletamine/zolazepam (Zoletil® 100 mg i.m.) and propofol (Rapinovet® 30 mg i.v.). All animals were intubated and anesthesia maintained throughout the procedure using a mixture of isoflurane (1–2%) in oxygen/nitrous oxide. Unfractionated heparin (70 units/kg i.v.) was given at the start of the procedure. Access to the coronary arteries was achieved via the left femoral artery, using standard six French sheaths and coronary guiding catheters. A total of 2–3 stents were placed under fluoroscopic guidance in different coronary arteries (reference diameter 3–3.5 mm, avoiding excessive tortuosity and major bifurcations) in either the left anterior descending (LAD), left circumflex (LCx), or right coronary arteries (RCA). Stents were deployed at inflation pressures necessary to produce a stent to artery ratio of 1.2:1 (10–12 atmospheres). After sheath removal, the femoral artery was ligated and the leg wound closed and sutured. All animals were given buprenorphine (Vetergesic® 0.15 mg i.m.) to provide analgesia and ampicillin (Amfipen® 350 mg i.m.) for antibiotic cover immediately after the procedure. Animals were allowed to recover and received a normal diet, with supplementation of oral aspirin 75 mg daily and oral clopidogrel 75 mg daily for the duration of the study. All premature and unexpected deaths were examined by post-mortem, gross evaluation, and stent examination. Approval was granted by Strathclyde University Ethics Review Committee, and the investigation conformed to the Guiding Principles in the Care and Use of Animals.

### Pharmacokinetic Studies

Drug loading of succinobucol coated stents was quantified by *in vitro* elution of SucES in pure ethanol (*n* = 4), followed by HPLC analysis. To determine the *in vivo* release characteristics of succinobucol, SucES were deployed in six pigs, using the same techniques as previously described. Pigs were euthanized by a lethal dose of pentobarbital at 1 hr, 1, 3, 7, 14, and 28 days after stent implantation. Two stents were implanted in each animal into different coronary arteries, with the exception of the 1 hr time point where three stents were used. Stents were removed carefully from freshly isolated arterial segments and succinobucol in the surrounding artery wall and remaining on the stent was extracted into acetonitrile. Samples were chromatographed on a Sphereclone ODS (2) column (5-μm particle size, 150 × 4.6 mm [Phenomenex, UK]). Samples were injected using an autosampler and pump system (Gynotek 480) in 20-μl aliquots, at a mobile phase flow rate of 1 ml/min acetonitrile–water (92.5:7.5). The detector (Detector 432, Kontron Instruments, UK) output was measured at a wavelength of 242 nm.

### Efficacy Study

Nineteen pigs underwent stenting performed by a single cardiologist blinded to the treatment group using 2–3 stents selected randomly on the morning of the procedure. Two unexpected premature deaths occurred within 24 hr of the procedure, and these pigs were not included in the 28-day efficacy analysis. In both cases, post-mortem revealed occlusive stent thrombosis with no evidence of myocardial infarction. It was not possible to determine whether a particular stent was responsible due to the small number of events. No other clinical events occurred during the study. In total, 17 pigs completed the 28-day efficacy study and 41 stents were available for histological evaluation (BMS, *n* = 11 [five LAD; three LCx; three RCA]; SucES, *n* = 10 [four LAD; four LCx; two RCA]; RES, *n* = 10 [three LAD; three LCx; four RCA]; and SucRES, *n* = 10 [four LAD; four LCx; two RCA]). Four out of 17 pigs, each receiving three stents, received two stents from the same group; all other pigs received different stents from either two or three groups. The stented coronary artery segments were dissected from the heart and flushed with normal saline to remove non-adherent thrombus. The specimens were fixed in formal saline and dehydrated in pure acetone before resin embedding in glycol methacrylate (Technovit 8100, Kulzer). Six sections were obtained from the proximal to distal portion of the stent using a Buehler Isomet 1000 rotary saw and mounted on a glass slide. Sections were then ground and polished using a Buehler Metaserv grinder to reduce the thickness to 10 μm and provide a uniform surface for staining and microscopic evaluation. Sections were stained using hematoxylin–eosin and modified Carstairs' stain. Images were acquired using a Leica DM LB2 microscope and Leica DFC320 digital camera. Blinded histological analysis was performed using computerized morphometry software (Image-Pro Plus, Cybernetics) according to published methods [22] and detailed examination by a consultant pathologist. The injury score for each strut was determined [22] and a mean score for each artery was calculated. Neointimal thickness was calculated as the mean distance from each stent strut to lumen; neointimal area was calculated as stent area minus lumen area; diameter stenosis was calculated as 100 × (1 − lumen area/IEL area). Binary ISR was defined as ≥50% diameter stenosis. Stent endothelialization score was defined as the extent of the circumference of the arterial lumen covered by ECs and graded from 1 to 3 (1 = 25%; 2 = 25–75%; 3 = ≥75%). Inflammation was graded as 0, none; 1, scattered inflammatory cells; 2, inflammatory cells encompassing 50% of a strut in at least 25–50% of the circumference of the artery; 3, inflammatory cells surrounding a strut in at least 25–50% of the circumference of the artery. The intimal fibrin content was graded as 0, no residual fibrin; 1, focal regions of residual fibrin involving any portion of the artery or moderate fibrin deposition adjacent to the strut involving <25% of the circumference of the artery; 2, moderate fibrin involving >25% of the circumference of the artery or heavy deposition involving <25% of the circumference of the artery; 3, heavy fibrin deposition involving >25% of the circumference of the artery. All sections were examined for evidence of uncovered stent struts, and the presence of in-stent luminal thrombus was determined throughout the entire stent length.

### Cell Culture

Bovine pulmonary artery SMCs, obtained from a local abattoir, were seeded on to six well plates (up to passage 4) and allowed to grow to 90% confluence. Succinobucol or probucol was added to each well at concentrations (1–20 μmol/L) that have previously been shown to inhibit SMC proliferation [23]. An additional well received the maximum amount of vehicle (0.3% dimethyl sulfoxide). After 24 hr, the well plates were inspected and photographed, taking account of any evidence of cytotoxicity. The medium was removed, and adherent cells were dislodged using trypLE Express (Invitrogen, UK). Trypan blue (0.07%) was added to the cell suspension and cells counted using a hemocytometer. The percentage of the counted cells not stained by trypan blue is reported as the percent viable cells. Identical experiments were performed using abattoir-derived bovine pulmonary artery ECs, grown to 70% confluence, and tested with succinobucol. The effect of probucol on ECs was not investigated as endothelialization was complete after 28 days in the pig model, and our assumption was that the adverse effect of succinobucol was more likely to be on SMCs, and this was compared directly to the parent drug (probucol), which acted as a control. In additional experiments, SIN-1 0.2 μmol/L (3-morpholinosydnonimine, a peroxynitrite donor) was used to generate oxidative stress and administered to bovine aortic SMCs and ECs alone and in combination with succinobucol or probucol.

### Statistical Analysis

Histomorphometric data for each stent were the mean of six sections from the proximal to distal end. The endothelial score, inflammatory score, and fibrin score were the mean of two sections per stent. Data were assessed for normality using the Shapiro-Wilk test. Normally distributed data are expressed as mean ± SD and groups compared using one-way analysis of variance with *post hoc* Dunnett's test. Non-parametric data are expressed as median (interquartile range) and groups compared using the Kruskal–Wallis test. Rates of binary restenosis were compared using the Fisher's exact test. Significance was established by a value of *P* < 0.05. For the efficacy study, a sample size of nine per group was calculated to provide 80% power to detect a treatment difference of 30% between groups at a two-sided 0.05 significance level, based on the assumption that the SD of the response variable (neointimal growth) was 20% as shown by similar studies in the porcine model [20]. Statistical analysis was performed using the SPSS statistical software package 14.0 for Windows (SPSS Inc., Chicago, IL, USA).

## RESULTS

### Pharmacokinetic studies

**Fig. 1:**
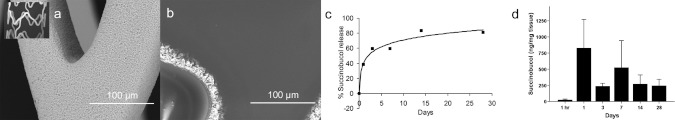
Characterization of succinobucol coated stents. (a) Expanded uncoated BMS showing surface modification allowing for drug deposition and release without the use of a polymer (inset, top left shows open cell stent design). (b) Expanded SucES showing smooth uniform 1% succinobucol coating, available for controlled release. (c) *In vivo* release profile of SucES. Succinobucol elution was controlled over 4 weeks, with the majority of drug released in the first week. Data points represent the mean of two measurements of mass of succinobucol released as a percentage of total drug loaded on the SucES (three stents were used for the 1 h time point). (d) Local succinobucol tissue concentration after SucES implantation. Succinobucol concentration peaked 1 day after implantation and was maintained for the duration of the study period. Data are mean ± SD using two identical stents implanted in the same pig (three stents were used for the 1 h time point).

The amount of drug loaded on the SucES using a 1% succinobucol coating solution was 465 ± 61 μg succinobucol per stent. The composition of succinobucol coating was assessed using scanning electron microscopy, which confirmed a smooth uniform drug layer, optimal for the Yukon DES delivery system (Fig. 1a and b). The drug loading of the 2% RES using prior data was 842.7 ± 46 μg per stent [21]. The SucES provided sustained *in vivo* drug release for 28 days (Fig. 1c); 59.4% of the total succinobucol loaded on the SucES was eluted during the first week and 81.0% was eluted after 28 days. Succinobucol concentration in coronary artery tissue immediately surrounding the stent quantified at 1 hr, 1, 3, 7, 14, and 28 days after stent implantation is displayed in Fig. 1d. Succinobucol tissue concentration peaked at 1 day post implantation (825.9 ± 631.3 ng/mg) and remained over 200 ng/mg during the remainder of the study. At 28 days, succinobucol tissue concentration was 242.2 ± 143.3 ng/mg. No succinobucol was detected in blood samples obtained from the pulmonary artery at 1 h or 1 day after stent deployment.

### *In Vivo* Efficacy

**Fig. 2:**
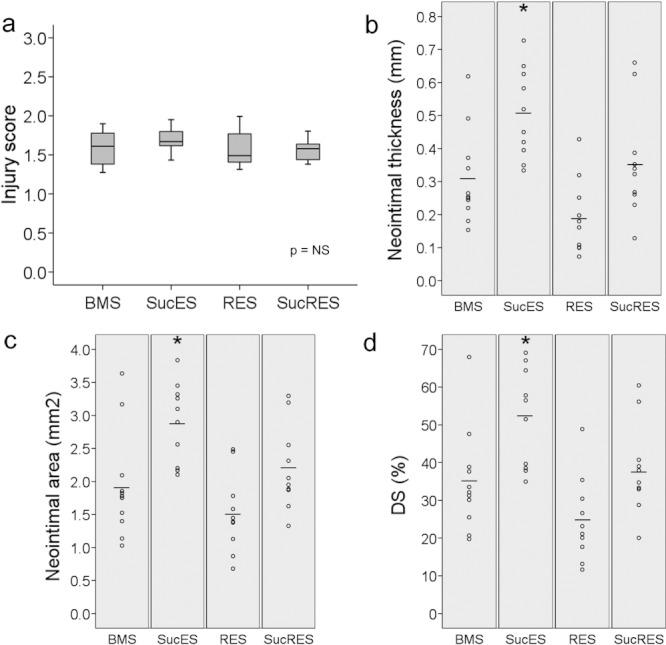
Histomorphometric data. (a) Box and whiskers plot showing injury scores, which were well matched between groups (*P* = 0.52). (b–d) Dot plots showing individual histomorphometric data. Lines represent group means. SucES caused a significant increase in neointimal thickening (b), neointimal area (c) and diameter stenosis (d) (*n* = 10–11, *P < 0.01 compared with BMS). The trends for RES to reduce neointimal thickening (*P* = 0.16) and diameter stenosis (*P* = 0.17) versus BMS were not statistically significant.

**Fig. 3:**
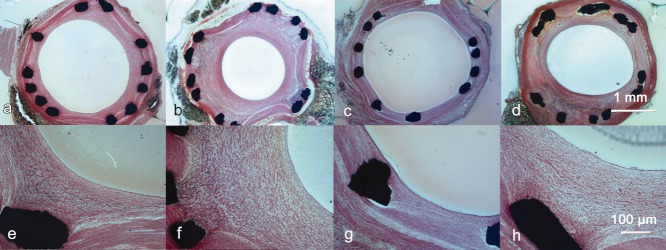
Representative photomicrographs of BMS (a, e), SucES (b, f), RES (c, g), and SucRES (d, h) groups. Neointimal thickening was significantly greater in the SucES group. The RES group displayed less neointimal thickening; however this was not statistically significant.

**Fig. 4:**
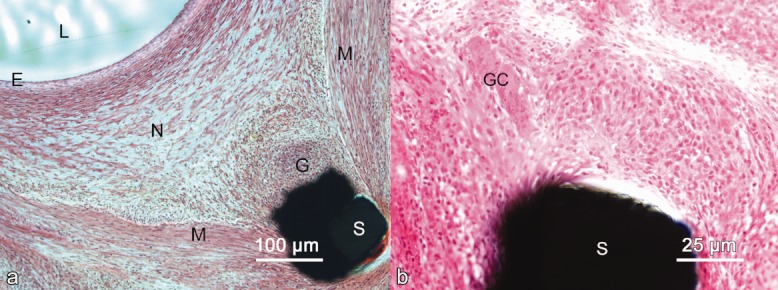
Histological findings in the SucES group. (a) Thick neointima (N) and stent strut (S) surrounded by inflammatory cells and macrophage granuloma (G). L, lumen; E, endothelial layer; M, medial layer. (b) Presence of a foreign body giant cell reaction (GC) adjacent to a stent strut (S).

The injury scores were well matched between groups (Table I and Fig. 2). Excessive injury (scoring 2 or more) was present in less than 20% of cases. Compared with BMS, SucES were associated with a significant increase in neointimal thickness, neointimal area, and diameter stenosis (Table I, Figs. 2 and 3). In all groups, the neointima was composed almost entirely of cells with little intercellular matrix. The rate of binary ISR measured by histological analysis was 9.1%, 60%, 0%, and 20% for BMS, SucES, RES, and SucRES groups, respectively (*P* < 0.01 between all groups). Histological findings related to healing and inflammation are shown in Table II. BMS and SucES were completely re-endothelialized at 28 days, whereas a non-significant reduction in endothelial regeneration was observed in both rapamycin groups (*P* = 0.21). The number of stents with any uncovered stent struts was 0/11 in BMS; 1/10 in SucES (one stent with 8.3% uncovered to total stent struts per section); 3/10 in RES (three stents with 25.0%, 16.7%, 10.0% uncovered to total stent struts per section), and 0/10 in SucRES groups (*P* = 0.08 between all groups). There were no cases of stent strut malapposition. The number of stents with in-stent thrombus was 27.3%, 10%, 20%, and 20% for the BMS, SucES, RES, and SucRES groups, respectively (*P* = 0.81). Fibrin scores were significantly higher in both succinobucol groups. Inflammation was increased in the SucES group and was characterized by predominantly lymphocytic infiltrates, with macrophage granuloma formation and foreign body giant cell reactions near to the stent struts in 6/10 stents (Fig. 4). In the two most severe cases of inflammation, minimal scattered eosinophils were present. The presence of granuloma formation and giant cells was identified to a much lesser extent in the BMS group (1/11 stents), whereas these findings were absent in the RES and SucRES groups, which were associated with milder lymphocytic infiltrates only. There was no significant difference in neovascularization, which was minimal in all groups.

**TABLE I:** Comparison of Histomorphometric Data at 28 Days

Group	Injury score	Neointimal thickness (mm)	Neointimal area (mm^2^)	Diameter stenosis (%)
BMS (*n* = 11)	1.61 (1.36, 1.82)	0.31 ± 0.14	1.93 ± 0.80	35.0 ± 13.6
SucES (*n* = 10)	1.67 (1.58, 1.82)	0.51 ± 0.14*	2.89 ± 0.61*	51.7 ± 13.1†
RES (*n* = 10)	1.49 (1.41, 1.78)	0.19 ± 0.11	1.52 ± 0.60	24.8 ± 11.2
SucRES (*n* = 10)	1.58 (1.43, 1.66)	0.36 ± 0.17	2.21 ± 0.64	38.4 ± 12.0

Values for injury score are median (IQR), other values are mean ± SD.

* *P* < 0.01 vs. BMS.

† *P* < 0.05 vs. BMS.

**Table II:** Healing and Inflammation

Group	Endothelial score	Fibrin score	Inflammatory score
BMS (*n* = 11)	3.0 (3.0, 3.0)	0.0 (0.0, 0.0)	2.0 (1.5, 2.0)
SucES (*n* = 10)	3.0 (3.0, 3.0)	1.0 (0.5, 1.1)*	2.3 (2.0, 2.6)†
RES (*n* = 10)	3.0 (2.9, 3.0)	0.0 (0.0, 0.5)	1.5 (1.5, 1.6)†
SucRES (*n* = 10)	3.0 (2.9, 3.0)	1.5 (0.4, 1.6)*	2.0 (2.0, 2.0)

All values are median (IQR).

* *P* < 0.005 vs. BMS.

† *P* < 0.05 vs. BMS.

### *In Vitro* Effects of Succinobucol on Cultured Endothelial and Smooth Muscle Cells

**Fig. 5:**
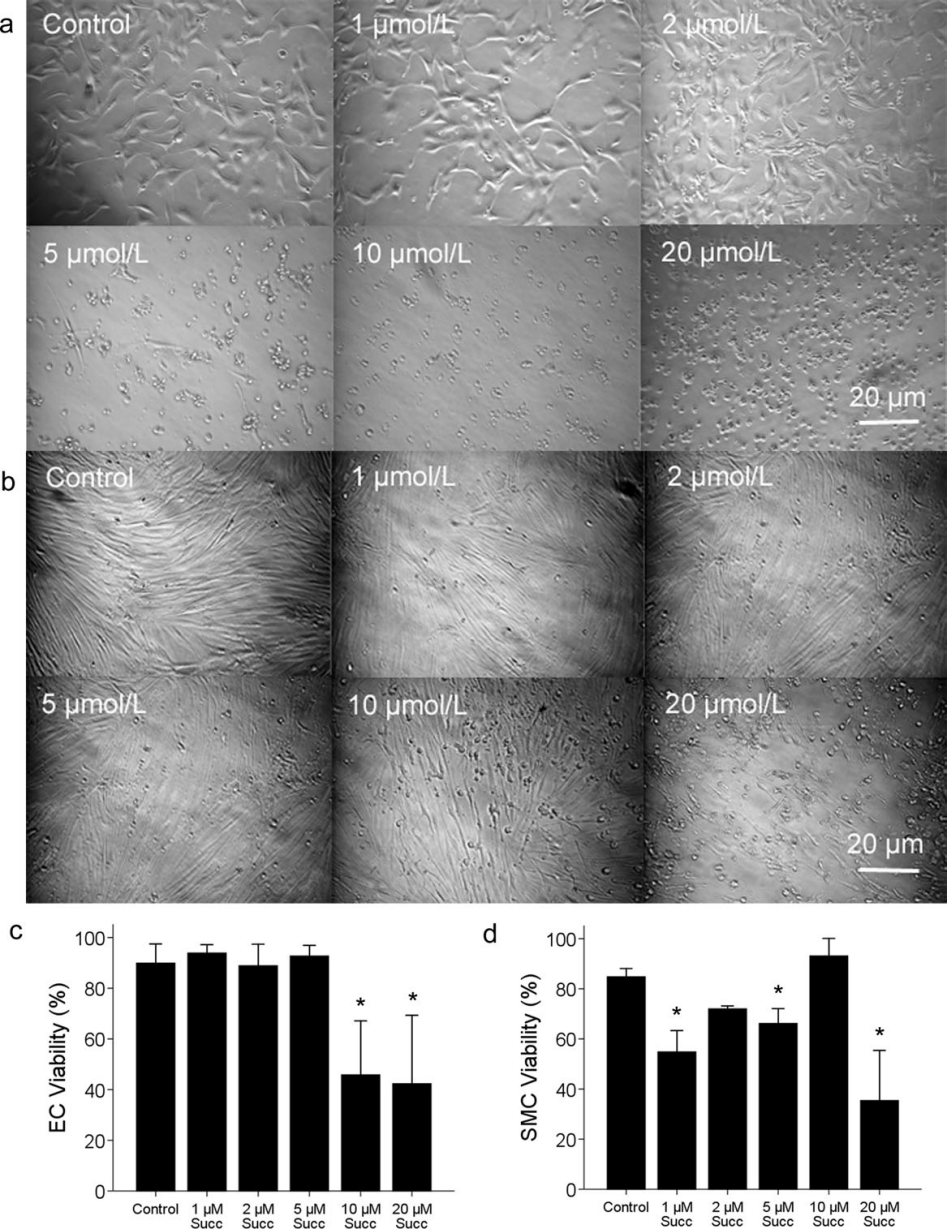
*In vitro* effects of succinobucol. ECs (a) and SMCs (b) in culture following 24 h incubation with succinobucol. There was evidence of concentration-dependent toxicity with a progressive reduction in cell adherence leading to total cell detachment at the highest concentration of succinobucol. The viability of the remaining adherent ECs (c) and SMCs (d) after 24 h incubation with succinobucol, showing further evidence of cellular toxicity. Values are mean ± SD (*n* = 4–6, *P < 0.05).

**Fig. 6:**
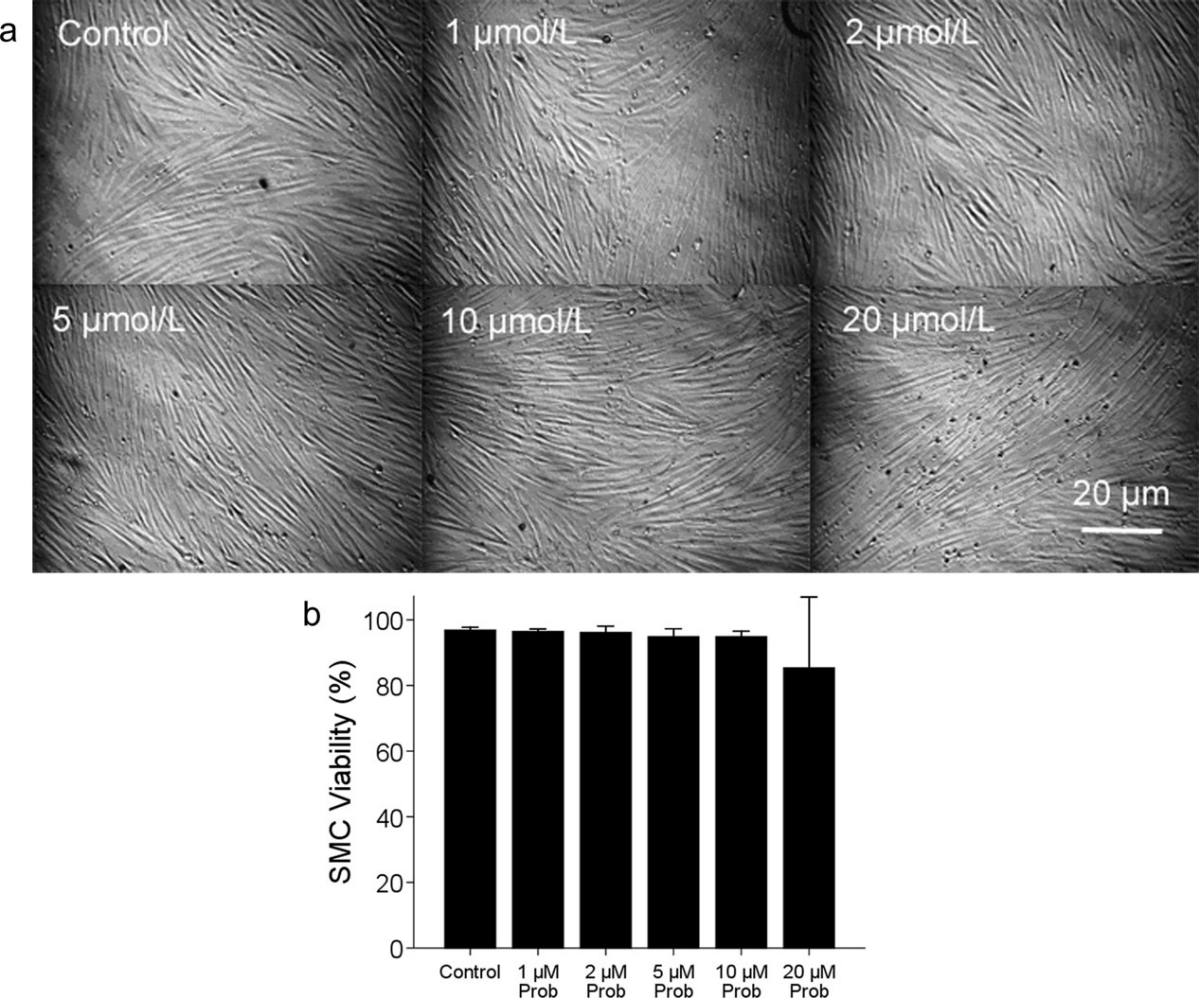
*In vitro* effects of probucol. After incubating SMCs (a) for 24 h with probucol, there was maintained cell adherence with increasing concentrations and only minor cell detachment at the highest concentration of probucol. (b) Probucol had no significant effect on the viability of remaining adherent SMCs. Values are mean ± SD (*n* = 4–5, *P* = NS).

**Fig. 7:**
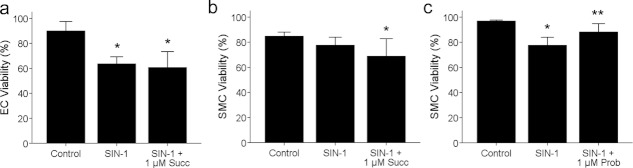
Succinobucol (a, b) failed to protect ECs and SMCs from SIN-1 induced toxicity, however probucol (c) provided some protection. Values are mean ± SD (*n* = 4–5, *P < 0.01 vs. control, **P < 0.05 for SIN-1 vs. SIN-1 + 1 μM probucol, *P* = NS all other comparisons).

Succinobucol caused toxicity to cultured bovine pulmonary artery ECs and SMCs; 24 h after addition of succinobucol 1 μmol/L, a few cells detached from the well plate in both EC and SMC cultures; whereas more than 10 μmol/L of succinobucol caused almost all cells to lift off the plate (Fig. 5a and b). Trypan blue staining showed that adherent ECs remained viable up to succinobucol 5 μmol/L; however, with higher concentrations, there was a decline in EC viability (Fig. 5c). The adherent SMCs had reduced viability at succinobucol 1, 5, and 20 μmol/L (Fig. 5d). Probucol was considerably less toxic to SMCs than succinobucol, with minor detachment of SMCs occurring only at 20 μmol/L (Fig. 6a). Probucol had no deleterious effect on SMC viability (Fig. 6b). SIN-1 0.2 μmol/L reduced the viability of ECs and SMCs. Succinobucol (1 μmol/L) did not protect against SIN-1-induced toxicity, whereas probucol provided some protection (Fig. 7).

## DISCUSSION

As both reactive oxygen species [24,25] and inflammation [26] are implicated in neointima formation, we chose to investigate the novel antioxidant and anti-inflammatory compound, succinobucol, without a polymer to reduce the potential for adverse effects. In our study, physical stent coating characteristics were optimal, and local delivery of succinobucol was well controlled, with around two-thirds of the drug released during the first week, matching closely the period after stent deployment when reactive oxygen species are released in large quantities [27]. This was very similar to the release profile of the RES, which retards drug release for greater than 3 weeks, with around two-thirds released during the first week [20,21]. However, stent-based delivery of succinobucol at this dose was found to increase neointimal growth and inflammation. Subsequently, we showed that succinobucol destabilizes cells in culture leading to cell detachment and loss of viability. However, cellular damage was much less marked with probucol. Thus, we postulate that succinobucol released from the stent induced direct cellular deterioration leading to the recruitment of inflammatory cells, as was observed *in vivo*. The presence of a widespread granulomatous reaction rich in lymphocytes, macrophages, and multinucleated giant cells is consistent with an unfavorable inflammatory response to the drug, given that these pathological findings were rare in the BMS group and mechanical injury scores were well matched. The extent of inflammation is known to correlate positively with neointimal growth [28], suggesting a direct link between increased inflammation and excessive neointimal thickening. The absence of eosinophils in the majority of artery specimens suggests that hypersensitivity did not play a major role. The persistent fibrin deposition identified around the stent struts *in vivo* also implies that succinobucol was responsible for impaired healing. Although antioxidants can provide protection against oxidative stress, they may also lead to generation of secondary radicals, which can modify important intracellular targets with the potential to cause cytotoxic effects [29,30]. This potential to act as pro-oxidants under certain conditions might explain some of the unfavorable cellular responses identified in our study. When used in combination with rapamycin, succinobucol impaired the antirestenotic effect of rapamycin. It is likely that the undesirable biological effects of succinobucol on the artery wall negated the favorable actions of rapamycin, although it is possible that succinobucol impeded the delivery of rapamycin.

### Comparison with Previous Studies

In CART-1, oral succinobucol improved coronary artery dimensions six months after stenting; however, it failed to influence neointimal growth, measured by intravascular ultrasound [9]. The ability of oral probucol to reduce neointimal growth is variable, with encouraging results in predominantly animal models [2–4,23,31], but negative reports in mainly human studies [6–9,32,33]. First-generation polymer-based DES are now recognized as being responsible for impaired healing and inflammation after coronary stenting [1,34]. Polymeric sirolimus-eluting stents are known to cause extensive granulomatous inflammatory reactions in the pig model, whereas persistent fibrin deposition is seen more commonly following exposure to polymeric paclitaxel-eluting stents [35,36]. Although difficulties remain in translating these and our findings to humans, these abnormal pathological responses may provide a substrate for late adverse clinical events. In support of this, post-mortem human studies have demonstrated impaired healing and increased arterial inflammation in cases of stent thrombosis following first-generation DES implantation [1,37,38]. Polymeric sirolimus-eluting stents also suffer from progressive restenosis between one and two years follow-up [12]. Thus, avoidance of a persistent inflammatory stimulus due to the stent, drug, or polymer remains a primary goal of novel DES to maintain late efficacy and safety. Notably, the effects of the dual SucRES in our study contrast with that of a polymer-free probucol/rapamycin stent on an identical platform, which has compared favorably to the sirolimus- and zotarolimus-eluting stents [12], with no increase in inflammation detected during preclinical assessment in pigs [10]. Therefore, stent-based delivery of succinobucol in combination with rapamycin clearly does not provide similar effects. Our study suggests that a critical factor determining the efficacy of antioxidants used in DES is the balance of their local cellular actions and toxicity, which may be dependent on a narrow therapeutic index. The one-third reduction in neointimal formation by the RES in our study was similar to previous preclinical data using an identical stent [20], which was later validated in clinical trials [21,39].

### Study Limitations

This investigation of succinobucol-coated stents after stent injury utilized a standard preclinical model. It is possible that the effect of local delivery of succinobucol in human atherosclerotic arteries would differ from the effect we have demonstrated in normal pig coronary arteries. A group consisting of BMS sprayed with solvent (99.5% ethanol) was not included; however, all coated stents were thoroughly dried with pressurized air by the coating machine prior to use; therefore a confounding effect of ethanol seems unlikely. To limit the number of groups, we chose to test a succinobucol dose similar to probucol in prior studies that also provided the most uniform stent coating. Lower concentration succinobucol solutions produced inferior strut coverage. The relationship between coating concentration, drug loading, and target tissue concentration is dependent on multiple factors including stent surface affinity for drug, *in vivo* release kinetics, and subsequent partitioning into tissues; therefore precise estimation of a coating concentration required to achieve a pre-specified local tissue concentration was not achievable. The local tissue concentration of succinobucol measured in our study exceeded the threshold for toxic effects *in vitro*. However, it is worth considering that the tissue concentration of rapamycin measured after successful drug elution in a porcine model [20] is around 1000-fold greater than that required to inhibit SMC proliferation [40], yet this stent is currently used in clinical practice. Further study would be required to determine the *in vivo* dose-response and long-term effects of succinobucol loaded on a stent; however, we think our results remain noteworthy and a beneficial action is unlikely.

## CONCLUSION

Our results suggest that 1% succinobucol is not a favorable compound for stent coating in clinical studies. There was increased neointimal formation and greater inflammation associated with the succinobucol groups after 28 days. The mechanism of this adverse effect may relate to local cell toxicity and resulting inflammation, or even pro-oxidant effects. The succinobucol coating also impaired the effect of rapamycin from a polymer-free DES, thereby reducing its antirestenotic properties. Future studies investigating novel antioxidants alone or in combination with other agents loaded on stents will require careful evaluation of their potential efficacy and local toxicity.
